# Metabolic trajectories in childhood and adolescence: Effects on risk for schizophrenia

**DOI:** 10.1038/s41537-022-00282-4

**Published:** 2022-10-11

**Authors:** Elina Sormunen, Maiju M. Saarinen, Raimo K. R. Salokangas, Nina Hutri-Kähönen, Jorma Viikari, Olli T. Raitakari, Jarmo Hietala

**Affiliations:** 1 Department of Psychiatry, University of Turku and Turku University Hospital, Turku, Finland; 2 Research Centre of Applied and Preventive Cardiovascular Medicine, University of Turku, Turku, Finland; 3 Tampere Centre for Skills Training and Simulation, Faculty of Medicine and Health Technology, Tampere University, Tampere, Finland; 4 Department of Medicine, University of Turku and Division of Medicine, Turku University Hospital, Turku, Finland; 5 Centre for Population Health Research, University of Turku and Turku University Hospital, Turku, Finland; 6 Department of Clinical Physiology and Nuclear Medicine, Turku University Hospital, Turku, Finland

**Keywords:** Schizophrenia, Psychosis

## Abstract

Abnormal glucose and lipid metabolism is common in antipsychotic-naive first-episode patients with schizophrenia, but it is unclear whether these changes can already be seen in premorbid or prodromal period, before the first psychotic episode. We examined insulin, total cholesterol, low-density lipoprotein (LDL) cholesterol, high-density lipoprotein (HDL) cholesterol, and triglyceride trajectories in children and adolescents (9–18 years old), who were later diagnosed with schizophrenia, any non-affective psychosis (NAP) or affective disorder (AD). The study population consisted of a general population-based cohort “The Cardiovascular Risk in Young Finns Study”, started in 1980 (*n* = 3596). Psychiatric diagnoses were derived from the Health Care Register up to the year 2018. Multivariate statistical analysis indicated no significant differences in insulin or lipid levels in children and adolescents who later developed schizophrenia (*n* = 41) compared to the cohort control group (*n* = 3202). In addition, no changes in these parameters were seen in the NAP (*n* = 74) or AD (*n* = 156) groups compared to the controls, but lower triglyceride levels in childhood/adolescence associated with earlier diagnosis of psychotic disorder in the NAP group. Taken together, our results do not support any gross-level insulin or lipid changes during childhood and adolescence in individuals with later diagnosis of schizophrenia-spectrum disorder. Since changes in glucose and lipid metabolism can be observed in neuroleptic-naive patients with schizophrenia, we hypothesize that the more marked metabolic changes develop during the prodrome closer to the onset of the first psychotic episode. The findings have relevance for studies on developmental hypotheses of schizophrenia.

## Introduction

Metabolic disturbances, such as abdominal obesity, low high-density lipoprotein (HDL) cholesterol, hypertriglyceridemia and diabetes, are common in patients with schizophrenia^1^. As much as one third of schizophrenia patients fulfill the criteria for metabolic syndrome^2^. The life expectancy of patients with schizophrenia is estimated to be 15–20 years shorter than with general population^3^ and a majority of the excess mortality is due to natural causes, especially cardiovascular diseases^4^. The use of antipsychotic medication has been consistently associated with weight gain^5^, insulin resistance^6^, hyperlipidemia^7^, and metabolic syndrome^8^. A part of these metabolic alterations may be the result of sedentary lifestyle^9^ or poor dietary habits^10^. However, there is evidence of robust alterations in several organ systems, including lipid and glucose dysregulation, in antipsychotic-naive first-episode psychosis patients, suggesting that at least some of the changes are related to the illness process itself^11^.

Diabetes-like glucose tolerance curves and abnormal responses to insulin have been linked to schizophrenia since the beginning of the twentieth century, well before the invention of antipsychotic drugs^12^. In fact, three large meta-analyses have shown wide disturbances in glucose metabolism in patients with first-episode psychosis, including elevated levels of fasting glucose and insulin, elevated levels of glucose after an oral glucose tolerance test and increased insulin resistance, irrespective of antipsychotic medication^13–15^. Similarly, according to further three meta-analyses, metabolic dysregulation in first-episode patients is not limited to glucose metabolism, but is also seen in lipid metabolism. All of the latter meta-analyses found lower levels of total cholesterol in patient with first-episode psychosis with no or minimal antipsychotic exposure^16–18^. Two of those meta-analyses also showed lower levels of LDL cholesterol and higher levels of triglycerides in first-episode psychosis patients^16,17^. Additionally, two meta-analyses found lower levels of HDL cholesterol in the same patient group^17,18^.

Despite convincing results of metabolic disturbances in established non-affective psychotic disorders, very few studies have been published addressing metabolic changes in premorbid or prodromal phase of schizophrenia. Our previous study showed that being underweight in childhood and adolescence (3 to 18 years of age) predicts later development of non-affective psychosis^19^. In 2008, Koponen et al.^20^ examined insulin resistance and lipid levels in 15–16 years old adolescents and found no significant difference between controls and 21 subjects who developed psychosis by the age of 20. However, two different studies have reported changes in specific aspects of the lipidome and proteome of 11 or 12 years old children, who later had psychotic experiences or developed psychosis identified by clinical interview^21,22^. Very recently Perry et al.^15^ showed that persistently high fasting insulin trajectory, at the age from 9 to 24 years, was also associated with the psychosis at-risk mental state and psychotic disorder.

Our aim was to study whether insulin and lipid trajectories differ from controls in children and adolescents who later develop schizophrenia, or any non-affective psychotic disorder defined by Diagnostic and Statistical Manual of Mental Disorders, 4th edition (DSM-IV). In relation to previous studies, our study design adds to the previous ones as we were able to perform repeated measurements of lipid and insulin levels in childhood and adolescence before the first psychotic episode. Additional advantages were, that the Care Register for Health Care was used for DSM-IV diagnostics and follow-up was conducted until the participants were over 40 years old, largely covering the risk age for non-affective psychoses.

## Methods

### Study sample

The study population is from the Cardiovascular Risk in Young Finns Study (YFS). YFS began in 1980 with a total of 3596 (83% of those invited) children and adolescents, from age groups of 3, 6, 9, 12, 15, and 18 years^23^. Participants were selected randomly, based on their social security numbers, from five Finnish cities (Helsinki, Turku, Tampere, Kuopio and Oulu) and nearby rural areas. Metabolic measurements used in this study were from first three follow-ups of 1980, 1983, and 1986, up to the participants’ age of 18 years (Supplement Table 1).

### Psychiatric outcome

Data on psychiatric diagnoses of participants was obtained from ﻿the Care Register for Health Care up to the year 2018. This register is maintained by the National Institute for Health and Welfare, and it covers all hospitals in Finland since 1969. ICD-diagnoses were converted to DSM-IV diagnoses in an identical way to our previous study^24^. We formed diagnostic groups of schizophrenia (DSM-IV 295), all non-affective psychoses including schizophrenia (DSM-IV 295, 297, 298), affective disorders (mood and anxiety disorders, DSM-IV 296, 300, 311), and controls with no psychiatric diagnoses related to hospital care. Each participant was categorized in only one diagnostic group with diagnoses prioritized in the order described above. Of the YFS cohort, 76 (2.1%) participants, 45 (59%) men and 31 (41%) women, were diagnosed with non-affective psychosis by the end of year 2018. Of those, 42 had schizophrenia, ﻿schizophreniform disorder, or schizoaffective disorder (DSM-IV 295), 6 had delusional disorder (DSM 297), and 28 had brief psychotic disorder or psychotic disorder NOS (DSM-IV 298). The prevalence for affective disorders (DSM-IV 296, 300, 311) was 4.7% (*n* = 169). Average age of first psychotic episode related to hospital treatment was 30 years (age ranged from 18 to 51).

The exclusion criteria were (1) psychiatric diagnose related to hospital care before the age of 19 (*n* = 13, one from schizophrenia group and 12 from affective disorders group), (2) type 1 diabetes mellitus before the age of 19 (*n* = 15, one from schizophrenia group, one from affective disorders group and 13 from controls), (3) a participant reporting unsuccessful fasting before insulin and lipid measurements or (4) a participant prohibiting the use of register data (*n* = 22). In addition, for insulin analyses, fasting insulin measurements of >200 mU/l were excluded. The number of participants with fasting blood samples at the age of 3 and 6 was low, especially in the schizophrenia group (*n* = 6–9). Physical activity index was only available from 9 years of age. Cut-off age for analysis of the premorbid or prodromal phase of psychosis was 18 years. Therefore, we used only age points of 9, 12, 15, and 18 years for all subjects. As the blood sample protocols were similar in years 1980–1986 and we have no reason to assume any remarkable differences between the birth cohorts, the follow-up series from study visits in 1980, 1983, and 1986, including children and adolescents from six age points, were combined for the analyses. After 1986 the next follow-up of the whole YFS cohort and metabolic measurements was done in 2001. This data is not used here as even the youngest participants were beyond our cut-off age. A total of 180 participants (8 from non-affective psychosis group, of which 2 with later schizophrenia, 15 from affective disorders group and 155 controls) had no fasting lipid or insulin measurements at the age points of 9, 12, 15, or 18 years. Final number of participants included in the analysis of 9 to 18 years old children and adolescents were as follows: 39 with later schizophrenia, 66 with any non-affective psychosis later in life, 141 with later affective disorder and 3047 controls.

Schizophrenia-spectrum is a heterogeneous group of disorders with variable clinical characteristics. We also explored whether childhood/adolescence insulin or lipid levels predict onset of psychosis in this sample. Follow-up times (lag-time) were defined as the difference between time of diagnosis and the last blood sample in the insulin or lipid trajectory (age ranged from 9 to 18 years). Time of diagnosis was defined as the date of first register entry of hospitalization for psychosis and used as a proxy for psychosis onset. Prodromal periods could not be defined in this type of register study. As precise dates of blood samples are not available in the data set, only the year of study visit, all follow-up times are scaled in full years. Controls were censored at the last check of registry data in 2018.

The protocol had the approval of the Ethics Committee of the Hospital District of Southwest Finland. Written informed consent was provided by all participants aged 9 and above and by the participants parents for younger children. The permissions for using register data and linking YFS data to diagnostic data were obtained from the respective organizations.

### Clinical characteristics

For the determination of serum lipid and insulin levels, venous blood samples were drawn in 1980, 1983, and 1986 after fasting overnight. Weight and height measurements were included in the physical examination during the follow-ups. BMI was calculated as kg/m^2^ and categorized as underweight vs. higher based on BMI cut offs for children and adolescents provided by Cole et al.^25^, underweight corresponding with adult BMI ≤ 18.5 kg/m^2^. Physical activity was estimated with physical activity index (PAI), based on a self-report questionnaire previously described in detail^24^. PAI was rated from 5 to 14 and a higher PAI represents higher amount of physical activity. Birthweight was asked from the participants’ parents in a questionnaire in 1983 and 1986 and classified to low birthweight (<2500 g) vs. higher. Parental mental disorders, diagnosed by a doctor, were asked in a questionnaire for participants parents in 1980 and 1983.

### Statistical methods

The descriptive statistics are given in each age point as *N* and mean (95% Cl) in different diagnostic groups and controls (Supplement Table 2). Heavily skewed distributions of serum triglycerides and insulin were log-transformed for the analyses; their data are given as geometric means. Associations of lipids and insulin with the risk of later schizophrenia, any non-affective psychosis or affective disorder are given as risk ratios with ﻿95% confidence intervals (RR [95% CI]) from univariate and multivariable modified Poisson regression models^26^. Generalized estimation equations were used in analyses of repeated measures^27^. All multivariable models included sex, age, low birthweight (≤2500 g), underweight, physical activity index and mother’s mental disorders as covariates. Mother’s mental disorders alone had a stronger effect on a participant’s risk for later non-affective psychosis than mental disorders of (a) father, (b) either parent, or (c) both parents^24^. Therefore, mother’s mental disorder was used as a covariate in the analysis. The multivariable models were further adjusted with age(time) x group interaction. All interaction terms proved to be non-significant (*p* > 0.05) and were excluded from the final analyses.

The follow-up times (in full years, from time of last blood sample to diagnosis) were analyzed with univariate and multivariable Cox regression analyses using the same covariates as in modified Poisson regression models above. Statistical analyses were done using SAS^®^ version 9.4 (SAS Institute, Cary, NC, USA) and IBM^®^ SPSS^®^ Statistics version 27 (IBM Corp., Armonk, NY, USA).

## Results

**Fig. 1:**
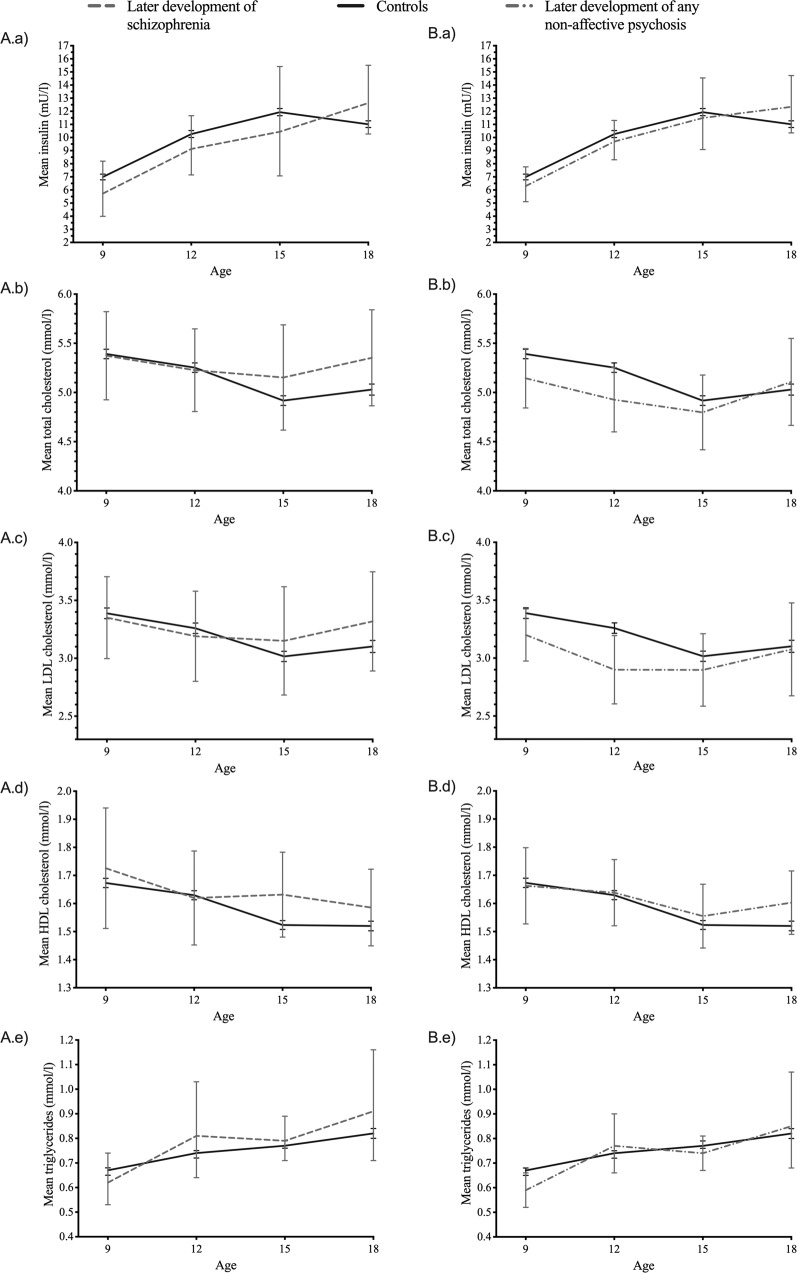
Childhood and adolescence lipid and insulin levels in participants who later developed schizophrenia, any non-affective psychosis and controls. Mean (95% CI) fasting plasma insulin^*^, total cholesterol, low-density lipoprotein (LDL) cholesterol, high-density lipoprotein (HDL) cholesterol and triglyceride* levels in children and adolescents (9–18 years of age) who later developed **A** schizophrenia^a^ or **B** any non-affective psychosis^b^ and controls. Gray dashed line = individuals who later developed schizophrenia, gray dash-dotted line = individuals who later developed any non-affective psychosis and black solid line = controls with no psychiatric diagnoses during the follow-up 1980–2018. *geometric means. ^a^DSM-IV diagnosis 295. ^b^DSM-IV diagnoses 295, 297, and 298.

At group level, there were no statistically significant differences in fasting lipid or insulin levels in childhood and adolescence between participants who were later diagnosed with schizophrenia or any non-affective psychosis and controls (Table 1, Supplement Table 2, and Fig. 1). Individual trajectories for participants with schizophrenia or any non-affective psychosis later in life are shown in Supplemental Fig. 1. Results were similar with patients who later developed affective disorder vs. controls (Supplement Table 3). The results on total cholesterol, HDL cholesterol, triglycerides, and insulin did not indicate even a trend for association between these parameters and risk for any studied diagnosis in this sample. LDL cholesterol seemed to be lower in children and adolescents with later non-affective psychosis especially at the age of 12. However, univariate analysis on whole trajectory from 9 to 18 years of age did not quite reach statistical significance (*p* = 0.054) and after adjusting for confounders in multivariate analysis *p*-values were >0.1 in all analyses.

**Table 1:** Childhood and adolescence fasting plasma insulin, total cholesterol, low-density lipoprotein (LDL) cholesterol, high-density lipoprotein (HDL) cholesterol and triglyceride levels at the age of 9 to 18 (1980–1986) and associated the risk of later development of schizophrenia^a^ or any non-affective psychosis^b^ up to the end of 2018.

Childhood and adolescence lipid and insulin levels	Risk of schizophrenia						Risk of any non-affective psychosis					
	Univariate			Multivariate^d^			Univariate			Multivariate^d^		
	RR	(95%Cl)	*p*	RR	(95%Cl)	*p*	RR	(95%Cl)	*p*	RR	(95%Cl)	*p*
1-unit lower insulin^c^	1.46	(0.8–2.7)	0.230	1.53	(0.8–2.8)	0.170	1.18	(0.8–1.9)	0.456	1.17	(0.7–1.9)	0.552
1-unit lower total cholesterol	0.89	(0.7–1.1)	0.338	0.89	(0.7–1.2)	0.403	1.25	(0.9–1.6)	0.124	1.17	(0.9–1.6)	0.239
1-unit lower LDL cholesterol	0.94	(0.7–1.2)	0.644	0.97	(0.7–1.3)	0.819	1.31	(0.99–1.7)	0.054	1.27	(0.95–1.7)	0.104
1-unit lower HDL cholesterol	0.61	(0.2–1.7)	0.341	0.50	(0.2–1.4)	0.196	0.82	(0.4–1.8)	0.612	0.62	(0.3–1.4)	0.239
1-unit lower triglyceride^c^	0.80	(0.4–1.4)	0.447	0.87	(0.5–1.6)	0.644	1.21	(0.8–1.9)	0.423	1.31	(0.8–2.1)	0.282

*RR* risk ratio; *CI* confidence interval.

^a^DSM-IV diagnosis 295.

^b^DSM-IV diagnoses 295, 297, and 298.

^c^Log-transformed in analyses.

^d^All multivariate analyses include sex, age, BMI underweight vs. higher, low (<2500 g) birthweight, physical activity index, and mother’s mental disorders.

We did additional exploratory analyses on lipid and insulin levels including only age points from 9 to 15 years (Supplement Table 4). Despite a trend for lower LDL cholesterol in those who later developed non-affective psychosis, all multivariate analysis results were statistically non-significant for non-affective psychoses and affective disorders.

Median lag-time from latest blood sample of the insulin/lipid trajectory to first psychotic episode was 17 years (range from 1 to 34 years) for non-affective psychosis group. For controls, median follow-up time in survival analysis was 32 years (range from 32 to 38 years). Multivariate analyses indicated that lipid and insulin levels did not associate with the psychosis onset in the schizophrenia and non-affective psychosis groups, but lower triglyceride level in childhood and adolescence was associated with earlier psychosis onset, i.e., shorter lag-time to diagnosis in the non-affective psychosis group (hazard ratio [95% CI] 2.2 [1.01–5.1], *p* = 0.047)) (see Supplement Table 5).

## Discussion

We found no significant differences in insulin or lipid levels between controls and children and adolescent who later developed schizophrenia, any non-affective psychosis or affective disorder. The results are in line with an earlier birth cohort study on lipid, glucose and insulin levels in adolescence (15–16 years) and later psychosis risk although the follow-up time in that study was short^20^. On the other hand, Perry et al.^15^ recently reported that persistently high fasting insulin level trajectory at the age of 9 to 24 years predicted a psychosis outcome at 24 years of age in the ALSPAC study. There are major design differences between the study of Perry et al. and the current study complicating the comparisons. First, the diagnostic procedures were different, i.e., semi structured psychosis-like experiences interview vs. formal ICD-diagnoses in psychiatry services in our study. Secondly, the follow-up time is different since Perry et al. are missing patients with psychosis onset after 24 years whereas follow-up in the current study covered well the risk periods for non-affective psychosis (up to 41–58 years of age). Nevertheless, it is certainly possible that there is a subtype of psychosis with early aberrant insulin levels, but this relatively rare subtype was not detected with our study design.

Several hypotheses have been proposed to explain the association between schizophrenia and metabolic disturbances. Some studies have suggested an overlapping genetic backgrounds of schizophrenia and cardiovascular risk factors^28,29^. However, a recent meta-analysis by Misiak et al.^30^ did not support this hypothesis, as they found no differences in the levels of fasting glucose, insulin or Homeostatic Model Assessment for Insulin Resistance (HOMA-IR) between first degree relatives of schizophrenia patients and controls. In fact, our findings together with the consistently observed metabolic dysregulation in neuroleptic-naive patients with first episode of non-affective psychosis suggest that the onset of changes in glucose tolerance and lipid metabolism is around the first episode. It is possible that the psychosis symptomatology, stress and other related, e.g., social factors induce an impairment in metabolic homeostasis resulting in glucose and lipid dysregulation^31^. These hypotheses need to be tested in future studies.

We were able to study only gross-level metabolic disturbances in premorbid and prodromal phases of psychotic disorders. Thus, more fine-grained changes in lipid or glucose metabolism as a part of non-affective psychosis trajectory are possible^21,22^. In this study total cholesterol and LDL cholesterol levels were lower in patients who later develop any non-affective psychosis than those of controls at age points of 9 and 12 years. Even though not statistically significant, this finding would be compatible with recent meta-analyses addressing lipids in first-episode psychosis^16–18^. Madrid-Gambin et al.^22^ showed that ﻿the lipidome and proteome are altered already at the age of 12 in subjects who report psychotic experiences at 18 years of age. Further, O’Gorman et al.^21^ found several lipids (lysophosphatidlycholines, phosphatidlycholines and sphingomyelin) significantly elevated in individuals who later developed psychosis at the age of 11, but not at the age of 18, suggesting ongoing alterations in the pathophysiological processes from prodrome to onset of psychosis. In our study, there was a trend for lower LDL cholesterol in children and adolescents (9–15 years), who later developed non-affective psychosis, but this difference was non-significant in the multivariate analysis. Also in our study, the difference in LDL-cholesterol levels between non-affective psychosis group and controls seem to disappear at the age point of 18. Thus, it is possible that there is a critical developmental window in prepuberty/puberty relevant for later metabolic disturbances and possible for a trajectory resulting in psychosis.

A recent study suggests that lipidomic abnormalities including structural triglyceride changes predate the onset of psychosis and could possibly be used to predict which clinical high-risk individuals are most likely to develop psychosis^32^. In our additional analyses, lower triglyceride levels in 9 to 18 years of age seemed to associate with earlier onset of psychosis. It is possible that patients with late onset psychosis have less metabolic alterations in childhood and adolescence or early metabolic abnormalities may relate to a specific type of non-affective psychosis with a relatively early onset of first psychotic episode. However, our triglyceride finding should be interpreted with caution, as it is may be affected by the relatively large number of covariates fitted in the data on relatively small number of participants in the psychosis group^33^. In any case it is evident that further studies are warranted on the more fine-grained lipid changes with possible etiological and clinical significance.

### Strengths and limitations

Our data is based on a non-selected and population-based cohort, which has a longitudinal and observational study design. Lipid and insulin levels were available from childhood to early adulthood, years before the first psychotic episode. Because of the study design, a full-scale follow-up was possible only for 6 years per participant. Thus, none of the subject’s data covers all follow-up points from 9 to 18 years of age, which limits detailed analyses of the individual metabolic trajectories throughout the study period. The number of participants is, nevertheless, relatively high in each time point, and we assume no significant difference between the age cohorts. Psychiatric diagnoses were obtained from the Care Register for Health Care, covering all hospital treatments of participants up to ages of 41–56 years. This is a strength in our study, considering that all except one previous study concerning lipid or insulin levels in childhood or adolescence and the risk of psychosis use clinical interviews to assess psychiatric outcomes and the follow-up for these outcomes was maximum 24 years of participant’s age^15,20–22^. The reliability of diagnosis of schizophrenia in the Care Register for Health Care has been reported to be high^34,35^. Studies have also shown that Finnish psychiatrists tend to apply a narrow definition of schizophrenia^36^. Therefore, in our study, specificity of diagnosis in schizophrenia group is likely to be very high, and we assume that some patients in the group of other non-affective psychosis actually have schizophrenia. The validity of affective disorder diagnoses in register-based studies have not been well documented and affective disorder patients requiring hospital treatment represent more severe forms of these disorders. As a limitation in our study, the number of participants who later developed psychosis, or especially schizophrenia, is relatively low. However, repeated lipid, insulin, and several other measurements already in childhood and low drop-out percentage still makes this sample informative.

## Conclusion

Knowledge about metabolic trajectories in childhood and adolescence, before first psychosis episode, is limited. Our group level results suggest that cholesterol, triglyceride, and insulin levels do not differ in children and adolescents with later development of schizophrenia, any non-affective psychosis or affective disorder compared to controls. Changes in triglyceride levels may modulate the time of psychosis onset but this hypothesis needs to be tested in further studies. We suggest that metabolic aberrations are indeed a part of early pathophysiological processes of schizophrenia-spectrum disorders but that the more marked changes in glucose and lipid metabolism may develop later, around the onset of first psychotic episode.

## Supplementary information

Supplement table 1

Supplement table 2

Supplement table 3

Supplement table 4

Supplement table 5

Supplement figure 1
