# Supplementary material for: Metabolic trajectories in childhood and adolescence: Effects on risk for schizophrenia
Source: Schizophrenia (Heidelb). 2022 Oct 11;8(1):82. doi: 10.1038/s41537-022-00282-4 (PMC9553975; doi:10.1038/s41537-022-00282-4)
Supplement: Supplementary file 1 — Supplement table 1 [file 41537_2022_282_MOESM1_ESM.pdf]

Supplement table 1. Fasting plasma insulin, total cholesterol, low-density lipoprotein (LDL) cholesterol, high-density lipoprotein (HDL) cholesterol and triglyceride levels, as well as participant's height and weight, were measured in 1980, 1983 and 1986. Physical activity index (PAI) was assessed in 1980, 1983 and 1986, parental mental disorders in 1980 and 1983 and birthweight in 1983 and 1986 by a questionnaire. In this study, we included measurements of participants aged 9 to 18 years. Measurements of 3- and 6-years old children were excluded due to low number of fasting blood samples and unavailability of physical activity index in children under 9 years old.

**Study design**									
**Year**	**Number of participants**	**Age cohorts**						**Metabolic measurements**	**Assessed by a questionnaire**
**1980**	3596	(3)	(6)	9	12	15	18	Height<br>weight<br>insulin<br>total cholesterol<br>LDL cholesterol<br>HDL cholesterol<br>triglycerides	PAI (from 9 years of age)<br>Parental mental disorders
**1983**	2991		(6)	9	12	15	18	Height<br>weight<br>insulin<br>total cholesterol<br>LDL cholesterol<br>HDL cholesterol<br>triglycerides	PAI (from 9 years of age)<br>Parental mental disorders<br>Birthweight
**1986**	2579			9	12	15	18	Height<br>weight<br>insulin<br>total cholesterol<br>LDL cholesterol<br>HDL cholesterol<br>triglycerides	PAI (from 9 years of age)<br>Birthweight
