# Supplementary material for: Metabolic trajectories in childhood and adolescence: Effects on risk for schizophrenia
Source: Schizophrenia (Heidelb). 2022 Oct 11;8(1):82. doi: 10.1038/s41537-022-00282-4 (PMC9553975; doi:10.1038/s41537-022-00282-4)
Supplement: Supplementary file 2 — Supplement table 2 [file 41537_2022_282_MOESM2_ESM.pdf]

Supplement table 2. N and mean (SD) of fasting plasma insulin, total cholesterol, low-density lipoprotein (LDL) cholesterol, high-density lipoprotein (HDL) cholesterol and triglyceride levels of children and adolescents (3 to 18 years old, during years 1980-1986) in the groups of later development of schizophrenia[a], any non-affective psychosis[b] or affective disorder[c] and controls with no psychiatric diagnoses during follow-up years 1980 – 2018. N refers to number of observations in each age point.

		**Total (n=3432)**	**Patients with schizophrenia (n=41)**			**Patients with non-affective psychosis (n=74)**			**Patients with affective disorder (n=156)**			**Controls (n=3202)**		
	Age	N	N	Mean	(95%Cl)	N	Mean	(95%Cl)	N	Mean	(95%Cl)	N	Mean	(95%Cl)
Insulin (mU/l)														
	3 years	562	6	4.9	(1.6–15.2)	13	4.7	(2.8–7.7)	34	3.5	(1.8–6.8)	479	2.9	(2.5–3.3)
	6 years	990	9	4.7	(2.5–8.8)	16	2.8	(0.6–12.3)	43	5.6	(4.9–6.4)	931	5.8	(5.6–6.0)
	9 years	1526	17	5.7	(4.0–8.2)	36	6.3	(5.1–7.8)	72	7.2	(6.3–8.2)	1418	7.0	(6.8–7.2)
	12 years	1574	19	9.1	(7.2–11.7)	33	9.7	(8.3–11.3)	65	11.1	(10.0–12.4)	1476	10.3	(10.0–10.5)
	15 years	1508	17	10.4	(7.1–15.4)	29	11.5	(9.1–14.5)	66	12.6	(11.1–14.3)	1413	11.9	(11.7–12.2)
	18 years	1282	14	12.6	(10.3–15.5)	19	12.3	(10.4–14.7)	48	11.3	(10.0–12.9)	1215	11.0	(10.8–11.3)
Total cholesterol (mmol/l)														
	3 years	531	6	5.0	(4.0–6.0)	13	5.5	(4.8–6.1)	34	5.2	(4.9–5.5)	484	5.2	(5.2–5.3)
	6 years	996	9	5.4	(4.5–6.3)	16	5.4	(4.9–5.9)	43	5.4	(5.2–5.7)	937	5.4	(5.4–5.5)
	9 years	1526	17	5.4	(4.9–5.8)	36	5.1	(4.8–5.4)	72	5.5	(5.2–5.7)	1418	5.4	(5.3–5.4)
	12 years	1578	19	5.2	(4.8–5.6)	33	4.9	(4.6–5.3)	65	5.2	(4.9–5.4)	1480	5.3	(5.2–5.3)
	15 years	1503	17	5.2	(4.6–5.7)	29	4.8	(4.4–5.2)	66	4.9	(4.7–5.1)	1408	4.9	(4.9–5.0)
	18 years	1282	14	5.4	(4.9–5.8)	19	5.1	(4.7–5.6)	48	4.9	(4.7–5.1)	1215	5.0	(5.0–5.1)
LDL cholesterol (mmol/l)														
	3 years	528	6	3.3	(2.4–4.2)	13	3.7	(3.2–4.2)	34	3.4	(3.2–3.7)	481	3.5	(3.4–3.5)
	6 years	992	9	3.5	(2.8–4.3)	16	3.5	(3.1–3.9)	43	3.5	(3.2–3.7)	933	3.5	(3.4–3.6)
	9 years	1521	17	3.4	(3.0–3.7)	36	3.2	(3.0–3.4)	71	3.5	(3.2–3.7)	1414	3.4	(3.3–3.4)

12 years	1571	19	3.2	(2.8–3.6)	33	2.9	(2.6–3.2)	64	3.2	(3.0–3.4)	1474	3.3	(3.2–3.3)
15 years	1496	17	3.2	(2.7–3.6)	29	2.9	(2.6–3.2)	66	3.0	(2.8–3.2)	1401	3.0	(3.0–3.1)
18 years	1279	14	3.3	(2.9–3.7)	19	3.1	(2.7–3.5)	47	3.0	(2.8–3.2)	1213	3.1	(3.1–3.2)
HDL cholesterol (mmol/l)													
3 years	528	6	1.4	(1.1–1.7)	13	1.5	(1.3–1.6)	34	1.4	(1.3–1.5)	481	1.5	(1.5–1.5)
6 years	992	9	1.6	(1.4–1.8)	16	1.6	(1.4–1.8)	43	1.7	(1.6–1.8)	933	1.6	(1.6–1.6)
9 years	1527	17	1.7	(1.5–1.9)	36	1.7	(1.5–1.8)	71	1.7	(1.6–1.7)	1420	1.7	(1.7–1.7)
12 years	1576	19	1.6	(1.4–1.8)	33	1.6	(1.5–1.8)	64	1.7	(1.6–1.7)	1479	1.6	(1.6–1.6)
15 years	1502	17	1.6	(1.5–1.8)	29	1.6	(1.4–1.7)	66	1.5	(1.4–1.6)	1407	1.5	(1.5–1.5)
18 years	1282	14	1.6	(1.4–1.7)	19	1.6	(1.5–1.7)	47	1.5	(1.4–1.6)	1216	1.5	(1.5–1.5)
Triglyceride (mmol/l)													
3 years	531	6	0.5	(0.3–0.8)	13	0.6	(0.4–0.7)	34	0.7	(0.6–0.8)	484	0.6	(0.6–0.6)
6 years	996	9	0.6	(0.4–0.8)	16	0.6	(0.5–0.7)	43	0.7	(0.6–0.7)	937	0.6	(0.6–0.7)
9 years	1530	17	0.6	(0.5–0.7)	36	0.6	(0.5–0.7)	72	0.7	(0.6–0.7)	1422	0.7	(0.7–0.7)
12 years	1578	19	0.8	(0.6–1.0)	33	0.8	(0.7–0.9)	65	0.7	(0.6–0.8)	1480	0.7	(0.7–0.8)
15 years	1506	17	0.8	(0.7–0.9)	29	0.7	(0.7–0.7)	66	0.8	(0.7–0.9)	1411	0.8	(0.8–0.8)
18 years	1283	14	0.9	(0.7–1.2)	19	0.9	(0.9–0.7)	48	0.8	(0.7–1.0)	1216	0.8	(0.8–0.8)

[a] DSM-IV diagnosis 295

[b] DSM-IV diagnoses 295, 297 and 298

[c] DSM-IV diagnoses 296, 300 and 311
