# Supplementary material for: Metabolic trajectories in childhood and adolescence: Effects on risk for schizophrenia
Source: Schizophrenia (Heidelb). 2022 Oct 11;8(1):82. doi: 10.1038/s41537-022-00282-4 (PMC9553975; doi:10.1038/s41537-022-00282-4)
Supplement: Supplementary file 3 — Supplement table 3 [file 41537_2022_282_MOESM3_ESM.pdf]

Supplement table 3. Childhood and adolescence fasting plasma insulin, total cholesterol, low-density lipoprotein (LDL) cholesterol, high-density lipoprotein (HDL) cholesterol and triglyceride levels at the age of 9 to 18 (1980–1986) and associated the risk of later development of affective disorder[a] up to the end of 2018. RR=Risk ratio; CI=confidence interval. *log-transformed in analyses.

**Childhood and adolescent lipid and insulin levels**	**Risk of affective disorder**					
	**Univariate**			**Multivariate****		
	RR	(95%Cl)	P	RR	(95%Cl)	P
1-unit lower insulin*	0.82	(0.6–1.1)	0.235	0.83	(0.6–1.1)	0.256
1-unit lower total cholesterol	1.04	(0.9–1.2)	0.672	1.06	(0.9–1.3)	0.501
1-unit lower LDL cholesterol	1.05	(0.9–1.3)	0.587	1.07	(0.9–1.3)	0.476
1-unit lower HDL cholesterol	0.98	(0.6–1.6)	0.923	1.03	(0.6–1.7)	0.917
1-unit lower triglyceride*	0.97	(0.7–1.4)	0.883	1.04	(0.7–1.5)	0.847

[a] mood and anxiety disorders, DSM-IV diagnoses 296, 300, 311

[**]All multivariate analyses include sex, age, BMI underweight vs higher, low (<2500g) birthweight, physical activity index, and mother’s mental disorders.
