# Supplementary material for: Metabolic trajectories in childhood and adolescence: Effects on risk for schizophrenia
Source: Schizophrenia (Heidelb). 2022 Oct 11;8(1):82. doi: 10.1038/s41537-022-00282-4 (PMC9553975; doi:10.1038/s41537-022-00282-4)
Supplement: Supplementary file 4 — Supplement table 4 [file 41537_2022_282_MOESM4_ESM.pdf]

Supplement table 4. Childhood and adolescence fasting plasma insulin, total cholesterol, low-density lipoprotein (LDL) cholesterol, high-density lipoprotein (HDL) cholesterol and triglyceride levels at the age of 9 to 15 (1980–1986) and associated the risk of later development of any non-affective psychosis[a] or affective disorders[b] up to the end of 2018. RR=Risk ratio; CI=confidence interval. *log-transformed in analyses.

**Childhood and adolescent lipid and insulin levels (age range 9-15 years)**	**Risk of any non-affective psychosis (DSM IV 295, 297, 298)**						**Risk of affective disorders (DSM IV 296, 300, 311)**					
	**Univariate**			**Multivariate****			**Univariate**			**Multivariate****		
	RR	(95%Cl)	P	RR	(95%Cl)	P	RR	(95%Cl)	P	RR	(95%Cl)	P
1-unit lower insulin*	1.32	(0.8–2.1)	0.246	1.29	(0.8–2.2)	0.329	0.82	(0.6–1.2)	0.255	0.80	(0.6–1.1)	0.210
1-unit lower total cholesterol	1.34	(0.98–1.8)	0.068	1.24	(0.9–1.7)	0.174	1.01	(0.8–1.2)	0.893	1.04	(0.8–1.3)	0.682
1-unit lower LDL cholesterol	1.39	(1.03–1.9)	0.034	1.33	(0.98–1.8)	0.065	1.03	(0.8–1.3)	0.813	1.06	(0.8–1.3)	0.622
1-unit lower HDL cholesterol	0.92	(0.4–2.3)	0.856	0.70	(0.3–1.7)	0.440	0.94	(0.6–1.5)	0.794	0.97	(0.6–1.6)	0.914
1-unit lower triglyceride*	1.31	(0.8–2.1)	0.284	1.34	(0.8–2.2)	0.268	1.007	(0.7–1.5)	0.972	1.02	(0.7–1.5)	0.918

[a] DSM-IV diagnosis 295, 297 and 298

[b] DSM-IV diagnoses 296, 300, 311

[**]All multivariate analyses include sex, age, BMI underweight vs higher, low (<2500g) birthweight, physical activity index, and mother’s mental disorders.
