# Supplementary material for: Metabolic trajectories in childhood and adolescence: Effects on risk for schizophrenia
Source: Schizophrenia (Heidelb). 2022 Oct 11;8(1):82. doi: 10.1038/s41537-022-00282-4 (PMC9553975; doi:10.1038/s41537-022-00282-4)
Supplement: Supplementary file 5 — Supplement table 5 [file 41537_2022_282_MOESM5_ESM.pdf]

Supplement table 5. Associations of childhood and adolescence fasting plasma insulin, total cholesterol, low-density lipoprotein (LDL) cholesterol, high-density lipoprotein (HDL) cholesterol and triglyceride levels at the age of 9 to 18 (1980–1986) with diagnosis lag-times (years to first psychotic episode) of later development of schizophrenia[a] or any non-affective psychosis[b] up to the end of 2018. HR=hazard ratio; CI=confidence interval from Cox regression analyses. *log-transformed in analyses.

**Childhood and adolescent lipid and insulin levels**	**Association with lag-time of first psychotic episode in schizophrenia**						**Association with lag-time of first psychotic episode in any non-affective psychosis**					
	**Univariate**			**Multivariate****			**Univariate**			**Multivariate****		
	HR	(95%Cl)	P	HR	(95%Cl)	P	HR	(95%Cl)	P	HR	(95%Cl)	P
1-unit lower insulin*	1.16	(0.5–2.5)	0.719	1.23	(0.5–2.8)	0.619	1.03	(0.6–1.9)	0.928	1.23	(0.5–2.8)	0.619
1-unit lower total cholesterol	0.89	(0.6–1.2)	0.463	0.82	(0.6–1.2)	0.260	1.15	(0.9–1.6)	0.347	1.003	(0.7–1.4)	0.984
1-unit lower LDL cholesterol	0.91	(0.6–1.3)	0.609	0.85	(0.6–1.3)	0.406	1.18	(0.8–1.7)	0.335	1.04	(0.7–1.5)	0.821
1-unit lower HDL cholesterol	0.55	(0.2–1.7)	0.299	0.43	(0.1–1.4)	0.166	0.78	(0.3–1.9)	0.585	0.49	(0.2–1.2)	0.132
1-unit lower triglyceride*	1.13	(0.4–3.0)	0.797	1.36	(0.5–3.8)	0.558	1.64	(0.8–3.4)	0.189	2.28	(1.01–5.1)	0.047

[a] DSM-IV diagnosis 295

[b] DSM-IV diagnoses 295, 297 and 298

[**]All multivariate analyses include sex, age, BMI underweight vs higher, low (<2500g) birthweight, physical activity index, and mother's mental disorders.
