# Supplementary material for: Metabolic trajectories in childhood and adolescence: Effects on risk for schizophrenia
Source: Schizophrenia (Heidelb). 2022 Oct 11;8(1):82. doi: 10.1038/s41537-022-00282-4 (PMC9553975; doi:10.1038/s41537-022-00282-4)
Supplement: Supplementary file 6 — Supplement figure 1 [file 41537_2022_282_MOESM6_ESM.pdf]

Supplement figure 1. Individual trajectories of fasting plasma insulin, total cholesterol, low-density lipoprotein (LDL) cholesterol, high-density lipoprotein (HDL) cholesterol and triglyceride levels in 9 to 18 years old children and adolescents who later developed A) schizophrenia[a] or B) any non-affective psychosis[b]. Black line = mean of controls.

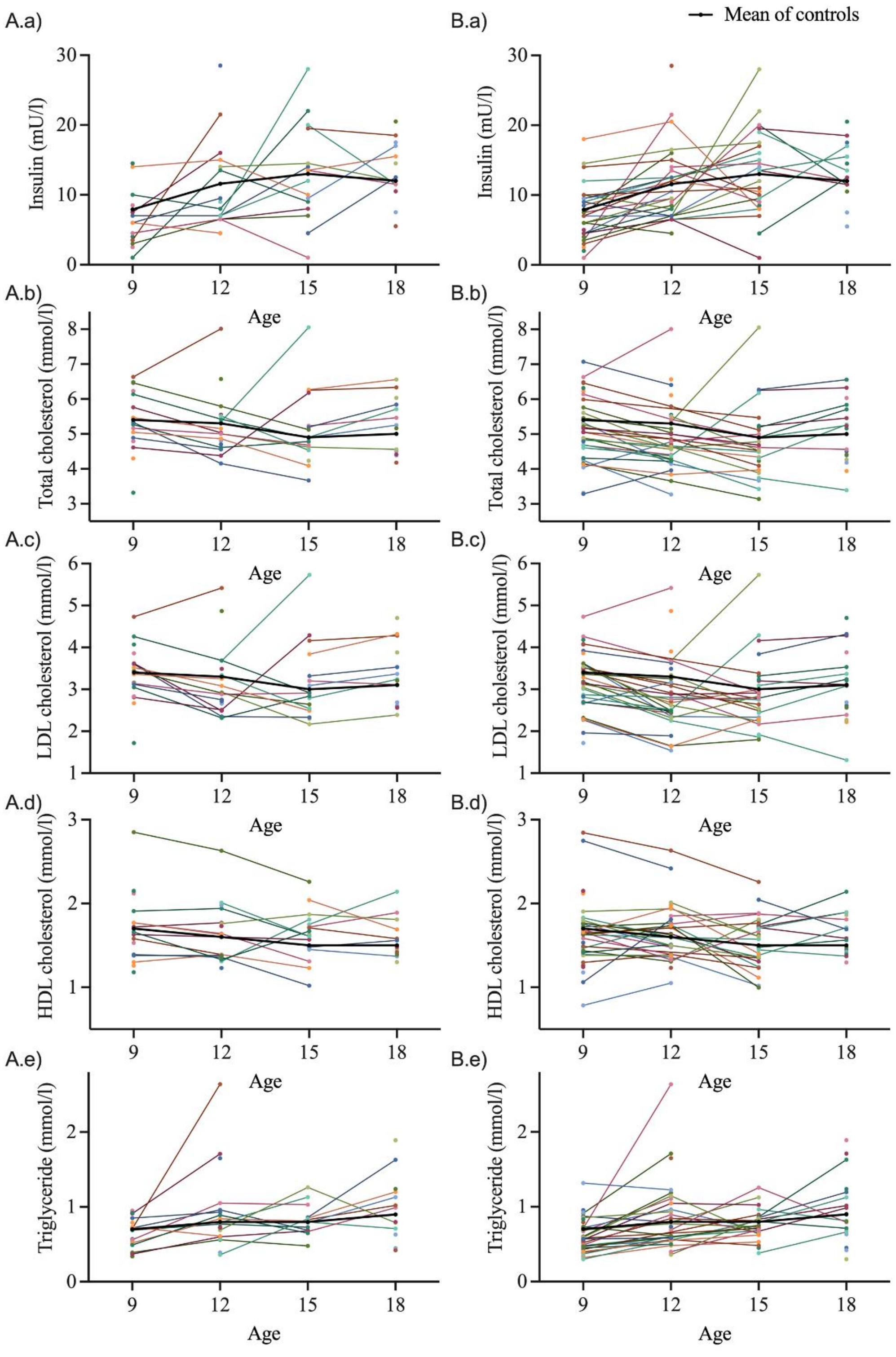

[a] DSM-IV diagnosis 295

[b] DSM-IV diagnoses 295, 297, 298
